# Supplementary material for: Hybrid Dynamic Pharmacophore Models as Effective Tools to Identify Novel Chemotypes for Anti-TB Inhibitor Design: A Case Study With Mtb-DapB
Source: Front Chem. 2020 Dec 2;8:596412. doi: 10.3389/fchem.2020.596412 (PMC7793862; doi:10.3389/fchem.2020.596412)
Supplement: Supplementary file 1 [file Data_Sheet_1.PDF]

Supporting Information

Hybrid dynamic pharmacophore models as effective tools to identify novel chemotypes for anti-TB inhibitor design: A case study with Mtb-DapB

Chinmayee Choudhury[1],

Anshu Bhardwaj[2*]

[1]Department of Experimental Medicine and Biotechnology, Post Graduate institute of Medical Education and Research, Sector 12, Chandigarh, India

[2]Bioinformatics Centre, CSIR-Institute of Microbial Technology, Sector 39A, Chandigarh, India

*Corresponding author

Email: anshu@imtech.res.in

Section S1. Details of MD simulation methodology

Molecular Dynamics (MD) Simulations

The Protein Preparation Wizard (PPW) module (Shivakumar et al., 2010) of Schrödinger software package, version 2019-2 was used to pre-process the macromolecular structure downloaded from PDB. Missing hydrogens were added, water molecules beyond 5 Å of the active site were removed and appropriate bond orders were assigned to the structure. The residues/side chains unresolved in some of the crystal structures were repaired with prime (Jacobson et al., 2002) module in the PPW. The protonation states of the polar residues were optimized with the protassign module of PPW, which uses PROPKA to predict pKa values (pH 7.0±2.0) and side chain functional group orientations. The structures were then subjected to restrained minimization (cutoff RMSD 0.3 Å) with impref to avoid steric clashes. These structures were further used for molecular dynamics (MD) simulations. MD simulations were carried out on the six model systems using the Desmond MD simulation package (release 2017) (Shaw et al., 2014). The OPLS_2005 (Jorgensen and Tirado-Rives, 1988) force field was employed for the protein-ligand complexes. Using the system builder tool of DESMOND the six model systems of Mtb-DapB were solvated in a cubical water box (TIP3P water model) keeping 10 Å buffer space in x, y and z dimensions. Each system was neutralized by adding appropriate counter ions and an ionic concentration of 0.15 M was maintained by adding Na+ and Cl− ions. The systems were minimized with 10000 steepest descent steps followed by gradual heating from 0 to 300 K, under NVT ensemble. The systems were thermally relaxed before the production run using Nose-Hoover Chain thermostat method for 1 picosecond and 1 picosecond of pressure relaxation with Martyna-Tobias-Klein barostat method. Finally, 0.2 microsecond (200 ns) production run under NPT ensemble was carried out for each system using a cutoff distance of 12 Å for non-bonded interactions. Simulation quality analysis, simulation event analysis, simulation interaction diagrams were used for trajectory analysis.

References

Jacobson, M. P., Friesner, R. A., Xiang, Z., and Honig, B. (2002). On the Role of the Crystal Environment in Determining Protein Side-chain Conformations. *J. Mol. Biol.* 320, 597–608. doi:10.1016/S0022-2836(02)00470-9.

Jorgensen, W. L., and Tirado-Rives, J. (1988). The OPLS [optimized potentials for liquid simulations] potential functions for proteins, energy minimizations for crystals of cyclic peptides and crambin. *J. Am. Chem. Soc.* 110, 1657–1666. doi:10.1021/ja00214a001.

Shaw, D. E., Grossman, J. P., Bank, J. A., Batson, B., Butts, J. A., Chao, J. C., et al. (2014). Anton 2: raising the bar for performance and programmability in a special-purpose molecular dynamics supercomputer. in *Proceedings of the International Conference for High Performance Computing, Networking, Storage and Analysis* SC '14. (New Orleans, Louisana: IEEE Press), 41–53. doi:10.1109/SC.2014.9.

Shivakumar, D., Williams, J., Wu, Y., Damm, W., Shelley, J., and Sherman, W. (2010). Prediction of Absolute Solvation Free Energies using Molecular Dynamics Free Energy Perturbation and the OPLS Force Field. *J. Chem. Theory Comput.* 6, 1509–1519. doi:10.1021/ct900587b.

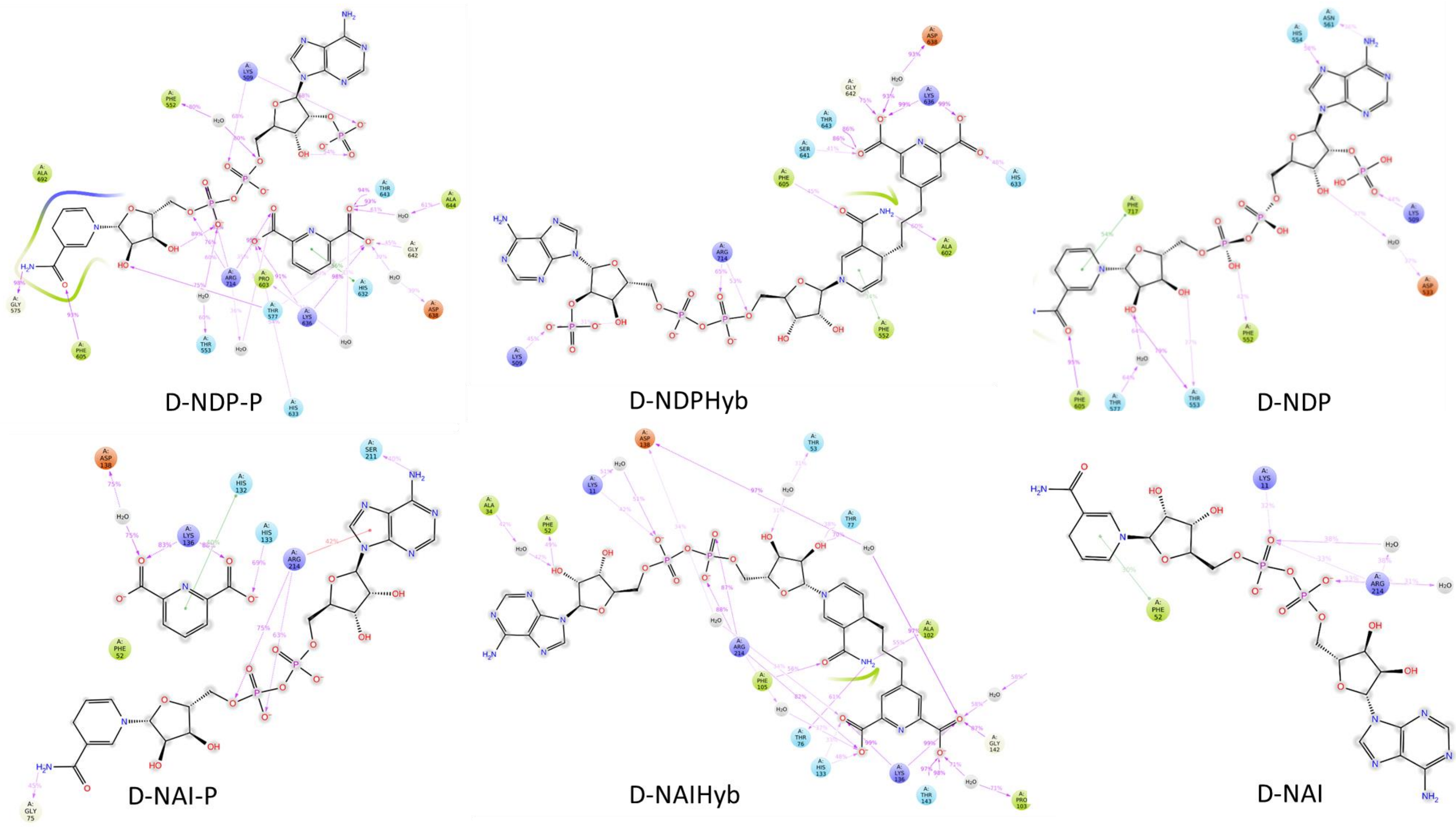

Figure S1. Stable protein-ligand interactions in the six model systems that lasted more than 30% of the simulation time.

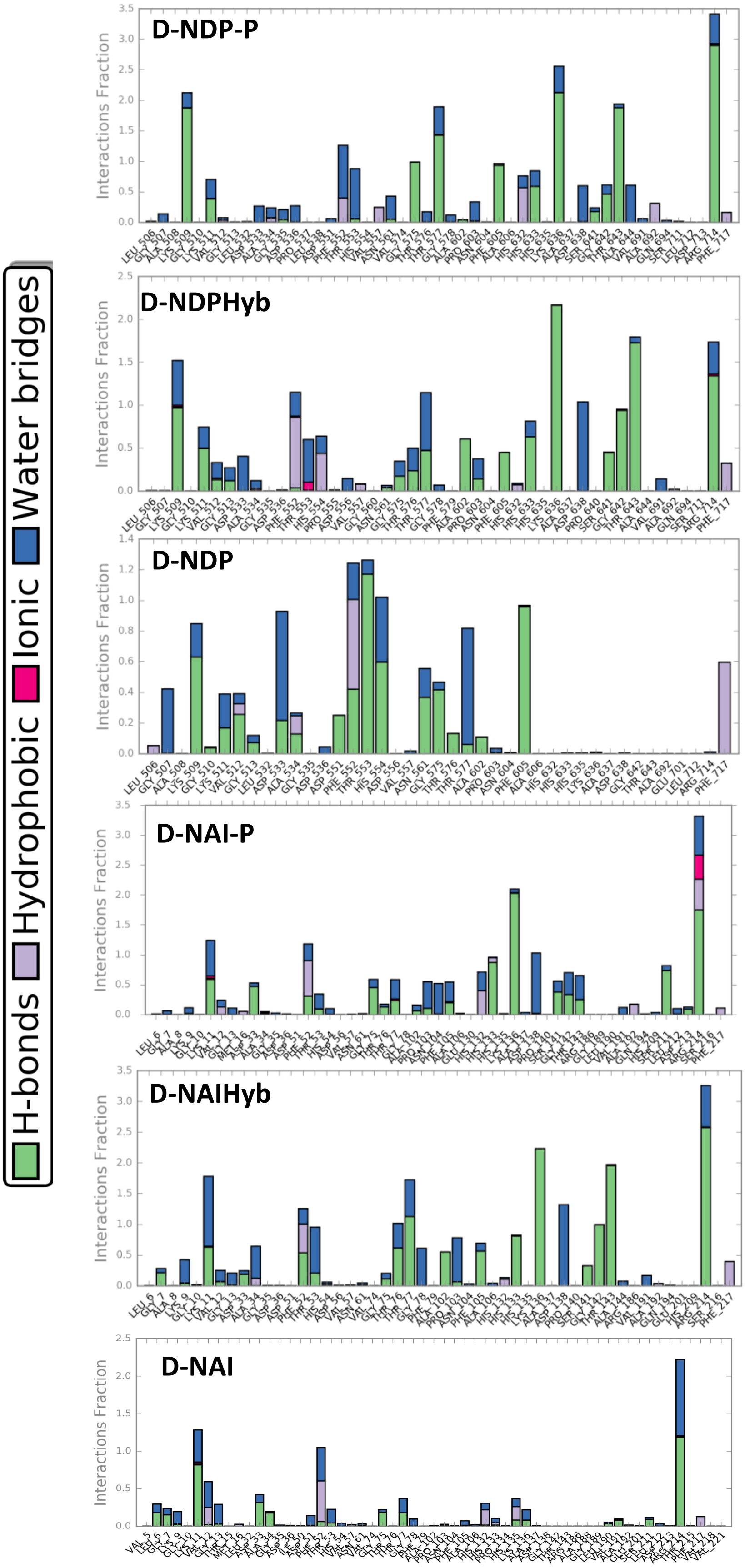

Figure S2. Fractions of different types of interactions made by the ligands with *Mtb*-DapB in the six model systems.

Table S1. Feature tables of all the six N-type pharmacophore models.

		**N-Type Model1 D-NDP-P (Cluster 1)**				
1	A11	-1.6	13.7992	33.9899	18.0959	HBond
2	N23	-0.69	18.3601	32.1812	14.6208	HBond
3	N22	-0.69	20.3436	28.4824	14.9029	HBond
4	R27	-0.67	13.8428	21.9542	16.029	RingChemscoreHphobe
5	D20	-0.65	14.6187	34.3047	23.6665	HBond
6	N25	-0.62	18.3972	22.7516	12.3691	HBond
7	A8	-0.33	18.2548	31.3989	16.7327	HBond
8	A12	-0.23	17.2656	35.5536	24.7898	HBond
		**N-Type Model2 D-NDP-P (Cluster 2)**				
1	A11	-3.20	30.46	9.00	-13.34	HBond
2	R27	-0.73	37.02	3.52	-5.87	Ring Chemscore
3	D20	-0.70	29.94	6.98	-17.89	HBond
4	N23	-0.69	27.73	7.65	-8.46	HBond
5	N22	-0.69	28.51	4.00	-6.07	HBond
6	N25	-0.62	32.57	5.64	-1.01	HBond
7	A12	-0.60	27.25	5.77	-18.99	HBond
		**N-Type Model3 D-NDP (Cluster 2)**				
1	D23	-1.6	25.1455	39.5989	40.6352	HBond
2	R30	-0.76	27.4781	34.7701	47.6988	RingChemscoreHphobe
3	A12	-0.7	19.4434	36.2985	36.3583	HBond
4	D24	-0.67	30.1244	33.9834	45.8677	HBond
5	A1	-0.08	27.9976	37.4251	46.8745	HBond
		**N-Type Model4 D-NDP (Cluster 4)**				
1	D22	-1.6	12.653	8.55	22.4881	HBond
2	A19	-1.33	12.6881	0.7402	29.684	HBond
3	R30	-0.85	17.7298	3.943	26.6276	RingChemscoreHphobe
4	A12	-0.7	8.0939	3.5405	15.7099	HBond
5	D27	-0.7	11.0348	4.5953	15.822	HBond
		**N-Type Model5 D-NDP (Cluster 6)**				
1	A20	-1.6	25.0936	-7.0556	-17.9801	HBond
2	A14	-1.03	19.5745	-7.1139	-10.2979	HBond
3	D22	-0.84	18.831	-1.8391	-12.4926	HBond
4	R30	-0.8	21.9953	-5.7205	-19.8722	RingChemscoreHphobe
5	D25	-0.7	20.6307	-3.7192	-21.9417	HBond
6	A12	-0.47	10.1189	-4.0745	-8.0887	HBond
7	D27	-0.16	11.0907	-2.2234	-10.3843	HBond
8	D21	-0.05	24.4512	-8.312	-14.9217	HBond
		**N-Type Model6 D-NAI-P (Cluster 1**				
1	N24	-1.91	5.0175	4.2839	-6.2929	HBond
2	D19	-0.67	12.6955	-0.3395	-9.8709	HBond
3	A2	-0.58	12.4315	2.112	-10.3629	HBond
4	H22	-0.52	9.883	10.3092	-6.9477	PhobEn
5	A8	-0.29	5.9034	5.5617	-7.9497	HBond
6	R25	-0.19	9.0993	2.2892	-10.3039	RingChemscoreHphobe

Table S2. Feature tables of all the six H-type pharmacophore models.

Rank	Feature_label	Score	X	Y	Z	source
**H-Type Model1 D-NDPHyb (Cluster 2)**						
1	N28	-1.85	18.82	46.6723	-6.4479	HBond
2	N29	-0.75	23.3865	42.902	-6.7673	HBond
3	D20	-0.7	13.9691	41.0351	-1.9275	HBond
4	N25	-0.66	23.4173	38.1076	-6.9987	HBond
5	R32	-0.46	20.6684	44.1271	-4.9795	RingChemscoreHphobe
6	A9	-0.21	21.5766	38.6976	-5.8601	HBond
**H-Type Model 2 from D-NDPHyb (Cluster 3)**						
1	A12	-2.44	-27.39	-12.27	-15.83	HBond
2	N29	-0.87	-29.56	-7.39	-20.94	HBond
3	N28	-0.65	-31.46	-5.61	-15.48	HBond
4	D21	-0.62	-24.75	-9.86	-12.77	HBond
5	A13	-0.51	-23.05	-7.36	-13.19	HBond
6	R32	-0.33	-28.82	-6.26	-17.72	Ring Chemscore
**H-Type Model 3 from D-NAIHyb (Cluster 1)**						
1	N27	-2.04	22.9181	24.1707	3.8691	HBond
2	A12	-1.6	24.0897	17.0952	8.524	HBond
3	N28	-1.2	19.9565	22.6982	8.763	HBond
4	D22	-0.62	23.4871	17.261	3.2131	HBond
5	R31	-0.55	20.219	22.5253	5.3252	RingChemscoreHphobe
**H-Type Model 4 from D-NAIHyb (Cluster 2)**						
1	A12	-1.60	2.38	-23.93	17.24	HBond
2	D17	-1.60	2.14	-26.73	14.84	HBond
3	N27	-1.25	1.90	-16.17	18.96	HBond
4	N28	-1.10	6.56	-19.80	19.04	HBond
5	D22	-0.70	-1.06	-21.93	19.50	HBond
6	R31	-0.59	3.72	-18.63	20.63	Ring Chemscore
**H-Type Model5 D-NDP-P (Cluster 1)**						
1	N5	-1.87	13.7992	33.9899	18.0959	HBond
2	A1	-1.6	14.6187	34.3047	23.6665	HBond
3	N6	-1.6	18.2548	31.3989	16.7327	HBond
4	D4	-0.65	17.2656	35.5536	24.7898	HBond
5	R7	-0.37	26.0106	13.0689	-17.513	RingChemscoreHphobe
6	A2	-0.33	23.2091	10.3234	-13.0278	HBond
7	A3	-0.23	24.4345	10.21	-16.2898	HBond
**H-Type Model6 D-NDP-P (Cluster 2)**						
1	A1	-3.2	30.46	8.9999	-13.339	HBond
2	N4	-2.18	20.963	36.9744	16.5854	HBond
3	N5	-1.36	15.4363	39.1187	17.9083	HBond
4	D3	-0.7	29.9351	6.98	-17.8893	HBond
5	R6	-0.66	18.9053	38.8579	18.71	RingChemscoreHphobe
6	A2	-0.6	27.2546	5.7702	-18.9887	HBond

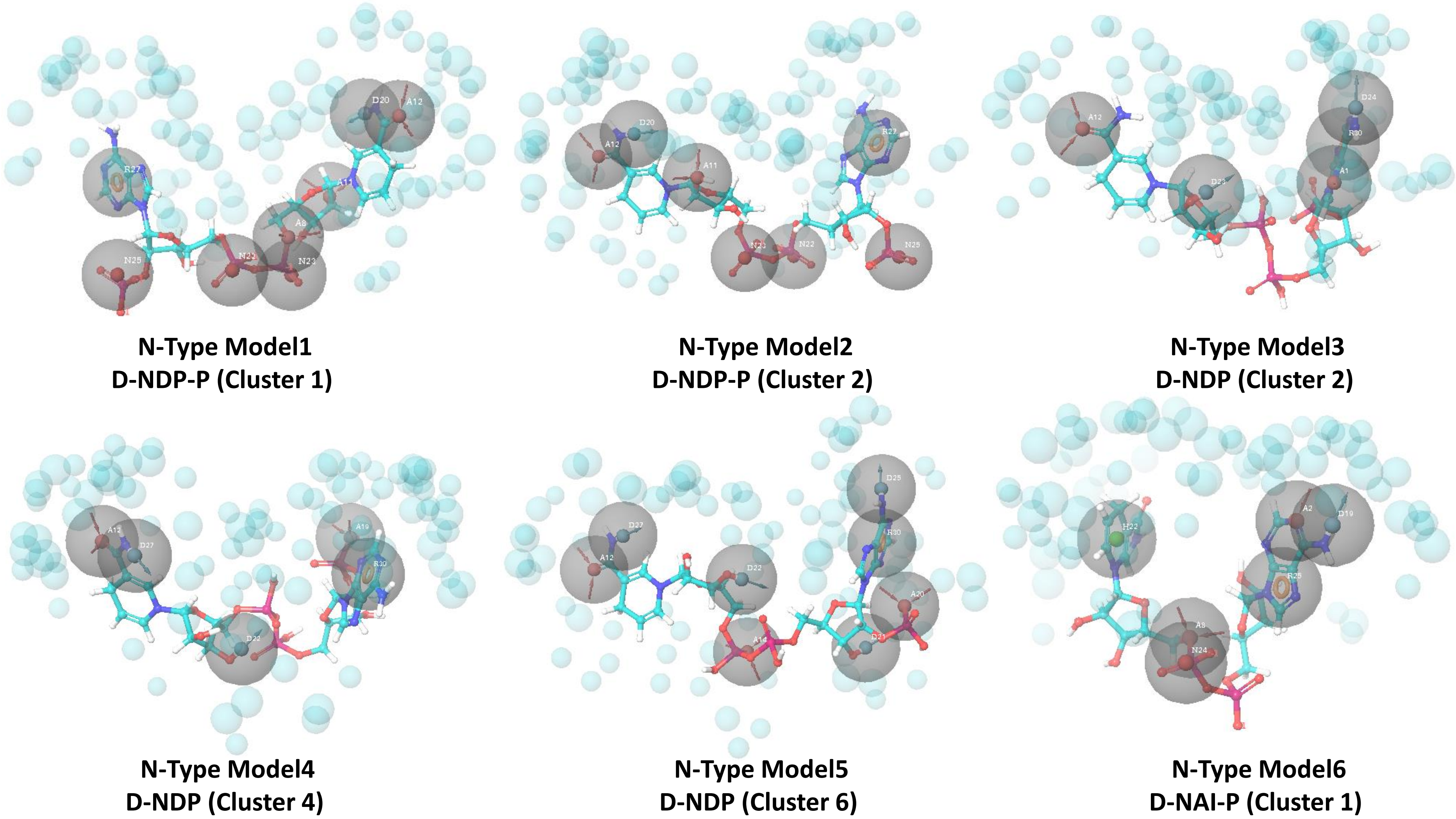

Figure S3. Snapshots of N-type pharmacophores with the cofactors aligned to them.

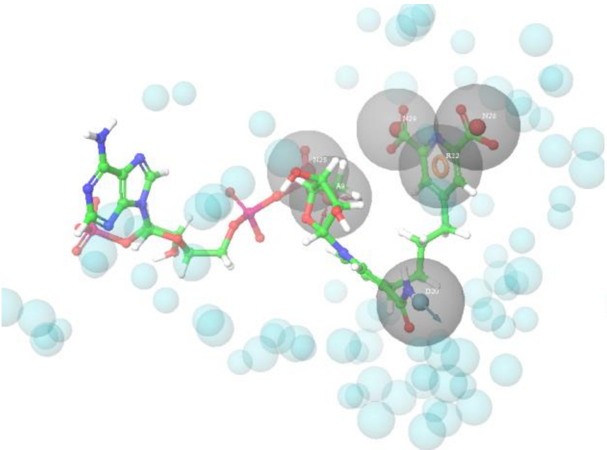

H-Type Model1
D-NDPHyb (Cluster 2)

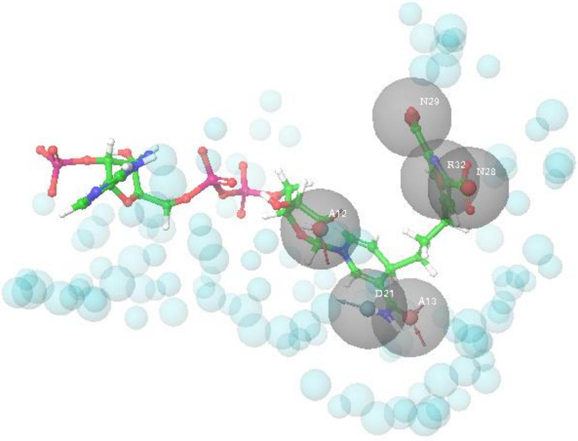

H-Type Model2
D-NDPHyb (Cluster 3)

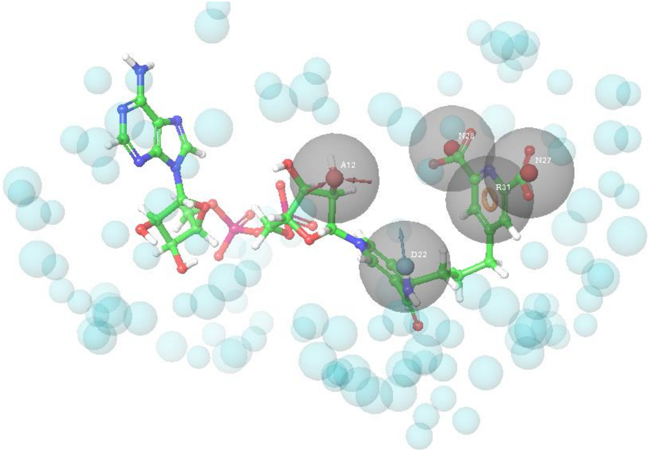

H-Type Model3
D-NAIHyb (Cluster 1)

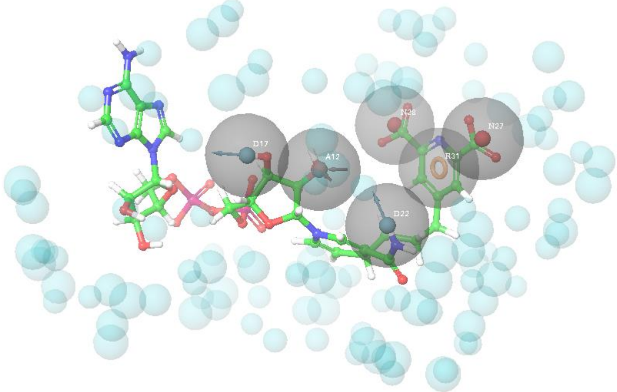

H-Type Model4
D-NAIHyb (Cluster 2)

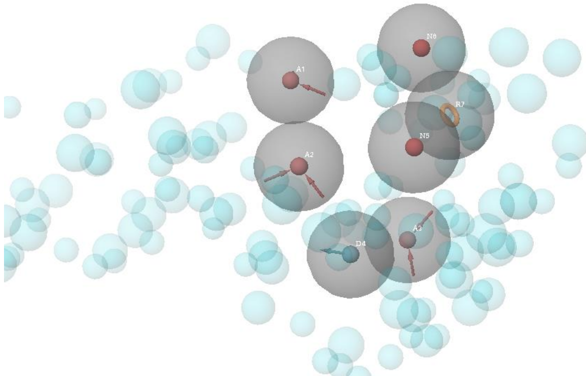

H-Type Model5
D-NDP-P (Cluster 1)

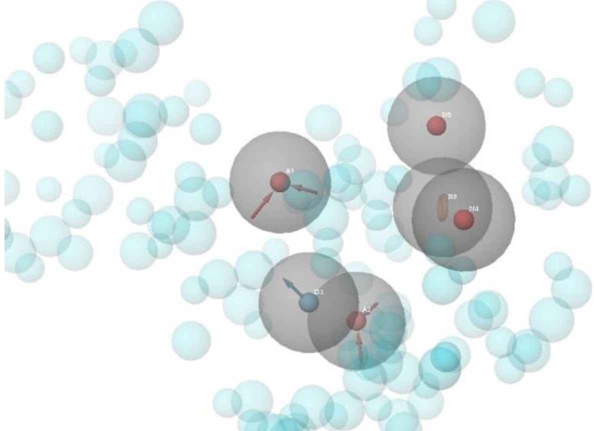

H-Type Model6
D-NDP-P (Cluster 2)

Figure S4. Snapshots of H-type pharmacophores with the first four aligned to the respective hybrid molecules. The last two are designed by combining two models, hence do not have a reference molecule.

Table S3 Screening results of Mtb-active and decoy molecules

Model	Total active molecules in the dataset	# of active molecules screened	Range of fitness score of the top screened active molecules	Total decoy molecules in the dataset	# of decoy molecules screened	Range of fitness score of the top screened decoy molecules	ROC
**H-type model**							
1	2349	225	0.59 to 0.71	12934	389	0.44 to 0.55	0.10
2	2349	815	0.63 to 0.71	12934	1205	0.31 to 0.65	0.35
3	2349	133	0.64 to 0.75	12934	236	0.40 to 0.58	0.06
4	2349	286	0.59 to 0.66	12934	505	0.26 to 0.54	0.13
5	2349	225	0.51 to 0.71	12934	372	0.22 to 0.53	0.10
6	2349	874	0.77 to 0.83	12934	1278	0.29 to 0.72	0.37
**N-type model**							
1	2349	22	0.39 to 0.79	12934	31	0.18 to 0.53	0.01
2	2349	39	0.33 to 0.76	12934	39	0.2 to 0.49	0.02
3	2349	13	0.38 to 0.72	12934	14	0.065 to 0.61	0.005
4	2349	1	0.37	12934	2	0.22 to 0.44	0
5	2349	1	0.33	12934	2	0.28 to 0.44	0
6	2349	22	0.26 to 0.63	12934	24	0.01 to 0.58	0.01

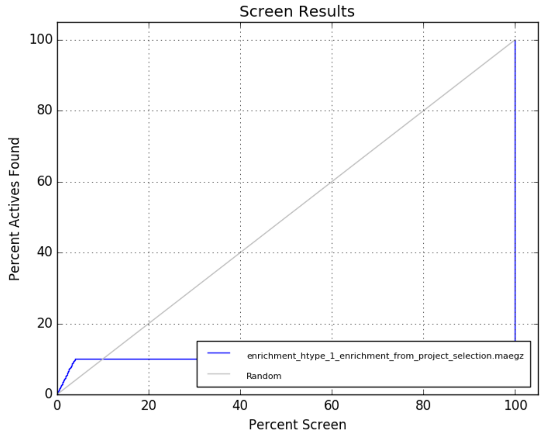

H-type model 1

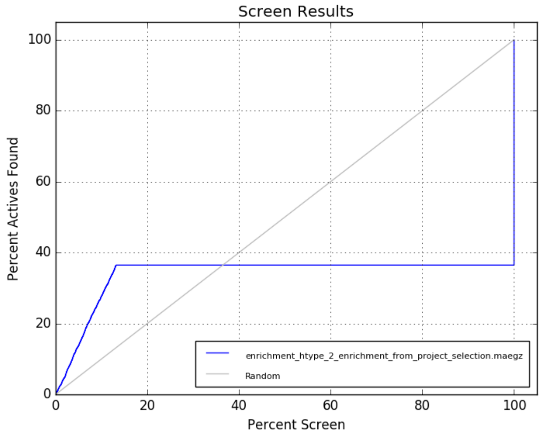

H-type model 2

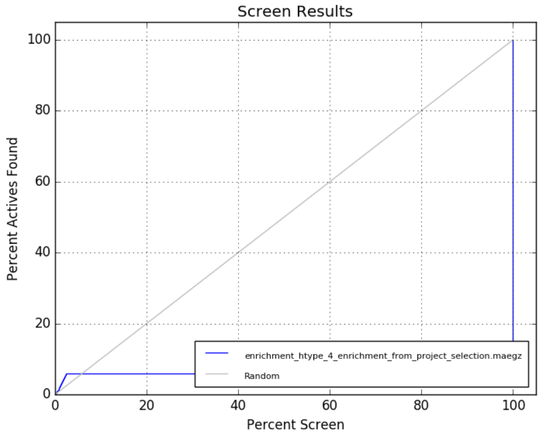

H-type model 3

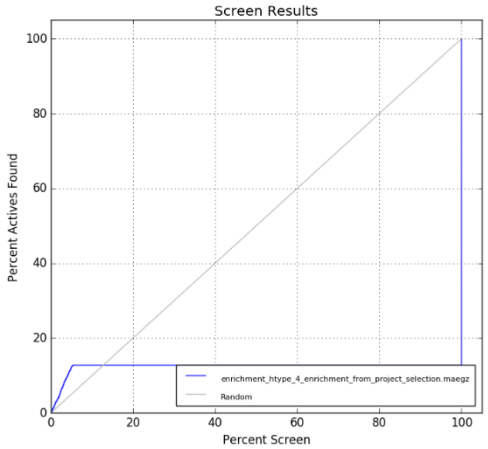

H-type model 4

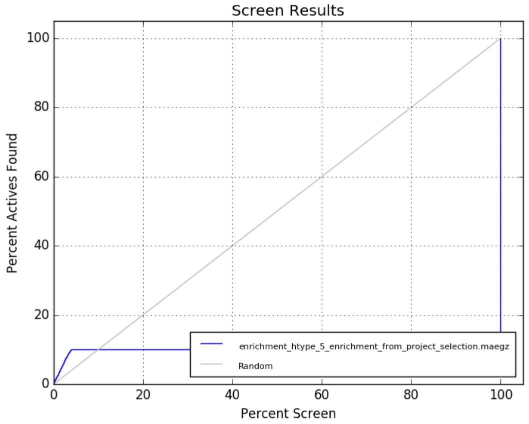

H-type model 5

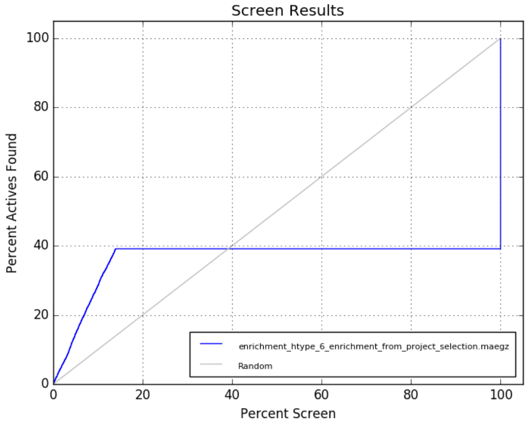

H-type model 6

Figure S5. ROC curves for Mtb-active and decoy molecules screened again H-type pharmacophore models

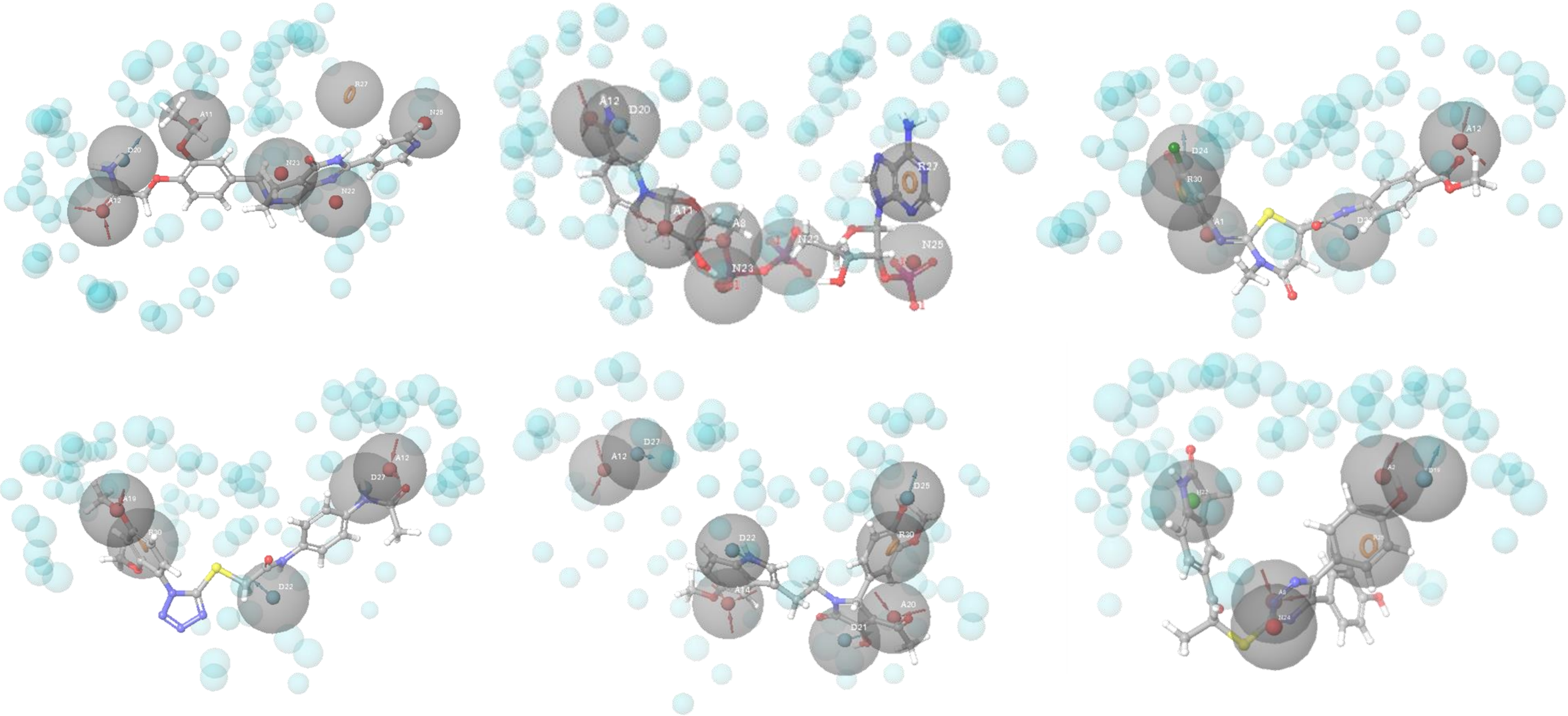

Figure S6. N-type pharmacophore models mapped to the respective top scoring compounds.

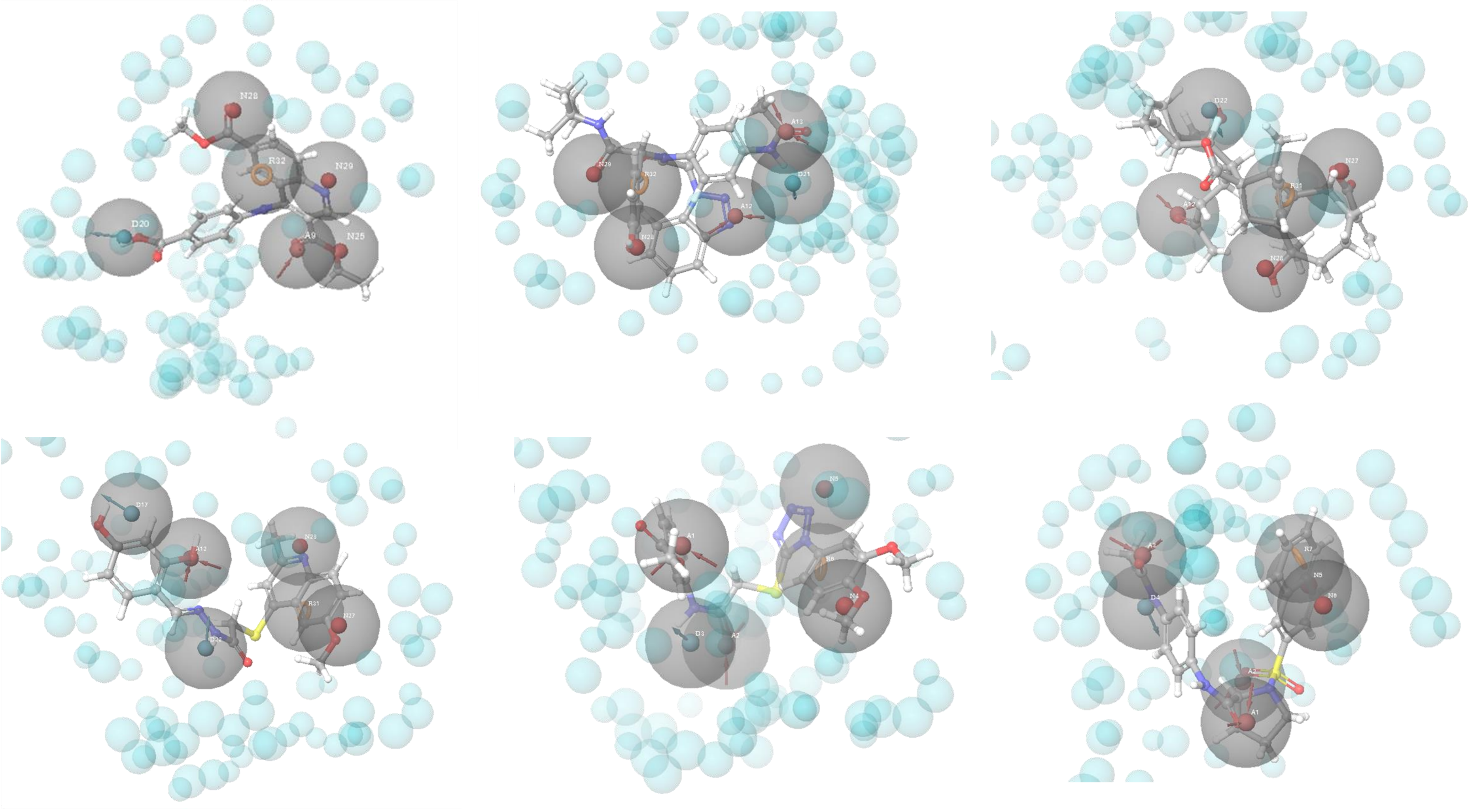

Figure S7. H-type pharmacophore models mapped to the respective top scoring compounds.

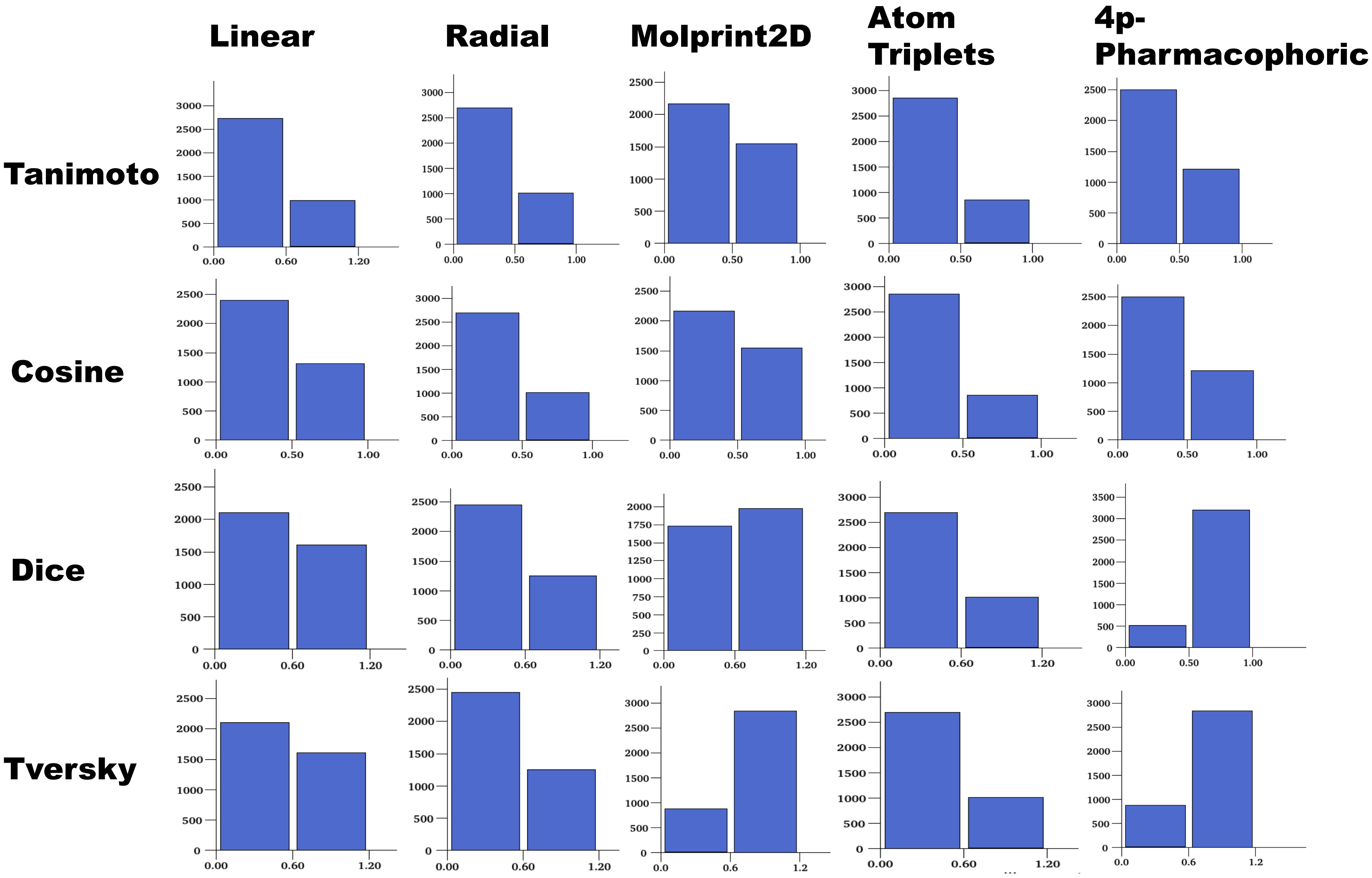

Figure S8. The similarity score distributions between H-set vs. N-set

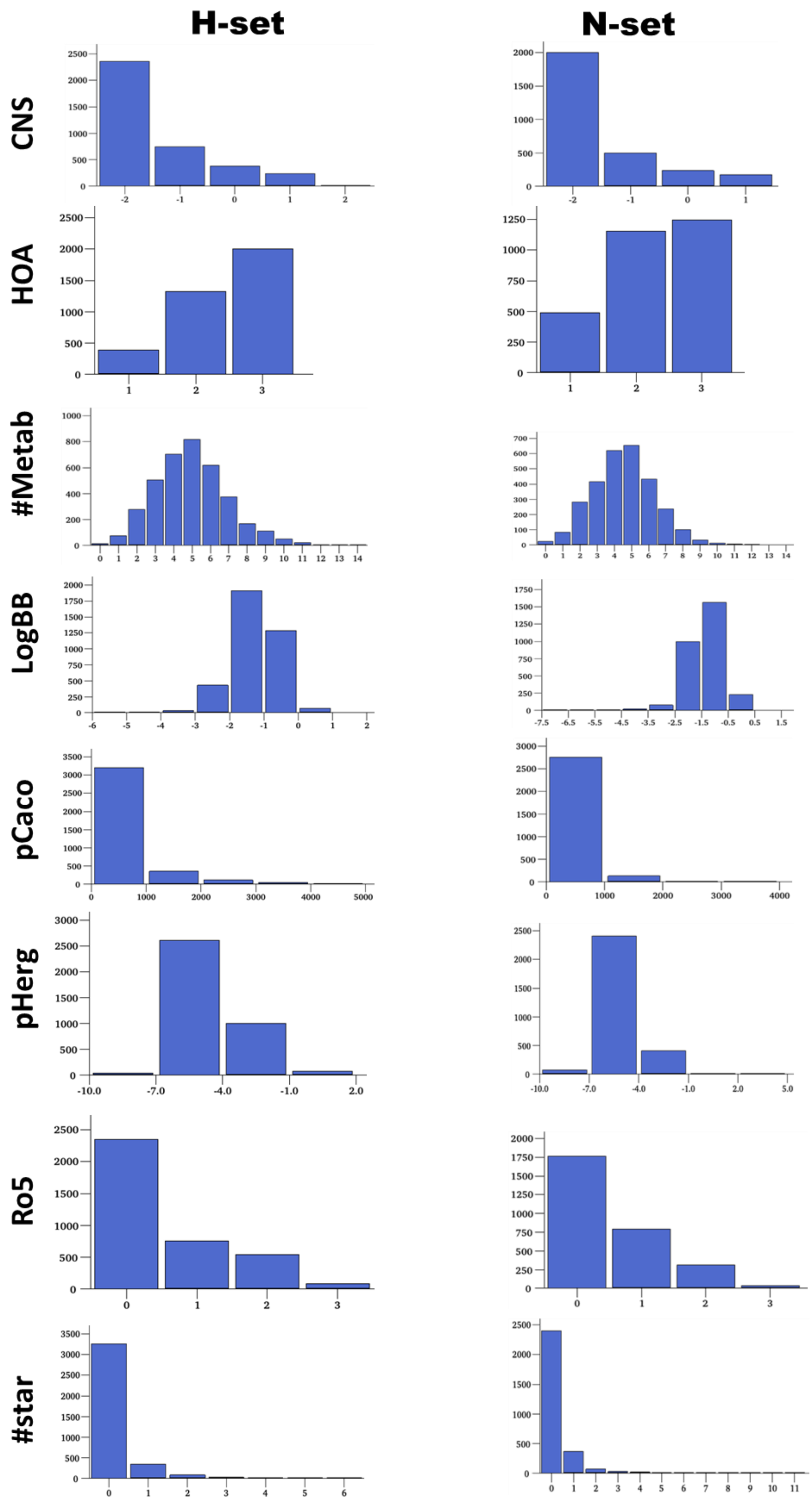

Figure S9. Distribution of different pharmacokinetic properties of the H-set and N-set compounds.

List S1. Details of all types QuikProp descriptors calculated in this study (recommended range of value for each property is mentioned in brackets).

• #stars Number of property or descriptor values that fall outside the 95% range of similar values for known drugs. The following properties and descriptors are included in the determination of #stars: MW, dipole, IP, EA, SASA, FOSA, FISA, PISA, WPSA, PSA, volume, #rotor, donorHB, accptHB, glob, QPpolrz, QPlogPC16, QPlogPoct, QPlogPw, QPlogPo/w, logS, QPLogKhsa, QPlogBB, #metabol (0 – 5)

• #rotor Number of non-trivial (not CX3), non-hindered (not alkene, amide, small ring) rotatable bonds. (0 – 15)

• mol_MW Molecular weight of the molecule. (130.0 – 725.0)

• SASA Total solvent accessible surface area (SASA) in square angstroms using a probe with a 1.4 Å radius. (300.0 – 1000.0)

• FOSA Hydrophobic component of the SASA (saturated carbon and attached hydrogen). (0.0 – 750.0)

• FISA Hydrophilic component of the SASA (SASA on N, O, and H on heteroatoms). (7.0 – 330.0)

• PISA (carbon and attached hydrogen) component of the SASA. (0.0 – 450.0)

• WPSA Weakly polar component of the SASA (halogens, P, and S). (0.0 – 175.0)

• volume Total solvent-accessible volume in cubic angstroms using a probe with a 1.4 Å radius. (500.0 – 2000.0)

• donorHB Estimated number of hydrogen bonds that would be donated by the solute to water molecules in an aqueous solution. Values are averages taken over a number of configurations, so they can be non-integer. (0.0 – 6.0)

• accptHB Estimated number of hydrogen bonds that would be accepted by the solute from water molecules in an aqueous solution. Values are averages taken over a number of configurations, sothey can be non-integer. (2.0 – 20.0)

• glob Globularity descriptor. (0.75 – 0.95)

• QPpolrz Predicted polarizability in cubic angstroms. (13.0 – 70.0)

• QPlogPC16 Predicted hexadecane/gas partition coefficient. (4.0 – 18.0)

• QPlogPoct‡ Predicted octanol/gas partition coefficient. (8.0 – 35.0)

• QPlogPw Predicted water/gas partition coefficient. (4.0 – 45.0)

• QPlogPo/w Predicted octanol/water partition coefficient. (–2.0 – 6.5)

• QPlogS Predicted aqueous solubility, log S. S in mol dm–3 is the concentration of the solute in a saturated solution that is in equilibrium with the crystalline solid. (–6.5 – 0.5)

• CIQPlogS Conformation-independent predicted aqueous solubility, log S. S in mol dm–3 is the concentration of the solute in a saturated solution that is in equilibrium with the crystalline solid. –6.5 – 0.5

• QPlogHERG Predicted IC50 value for blockage of HERG K+ channels. (concern below –5)

• QPPCaco Predicted apparent Caco-2 cell permeability in nm/sec. Caco-2 cells are a model for the gut-blood barrier. QikProp predictions are for non-active transport. (<25 poor, >500 great)

• QPlogBB Predicted brain/blood partition coefficient. (–3.0 – 1.2)

• QPPMDCK Predicted apparent MDCK cell permeability in nm/sec. MDCK cells are considered to be a good mimic for the bloodbrain barrier. QikProp predictions are for non-active transport. (<25 poor, >500 great)

• QPlogKp Predicted skin permeability, log Kp. (–8.0 – 1.0)

• QPlogKhsa Prediction of binding to human serum albumin. (–1.5 – 1.5)

• HumanOralAbsorption Predicted qualitative human oral absorption: 1, 2, or 3 for low, medium, or high.

• PercentHuman- OralAbsorption Predicted human oral absorption on 0 to 100% scale. The prediction is based on a quantitative multiple linear regression model. This property usually correlates well with HumanOralAbsorption, as both measure the same property. (>80% is high ,<25% is poor)

• PSA Van der Waals surface area of polar nitrogen and oxygen atoms. (7.0 – 200.0)

• RuleOfFive Number of violations of Lipinski's rule of five. The rules are: mol_MW < 500, QPlogPo/w < 5, donorHB ≤ accptHB ≤10. Compounds that satisfy these rules are considered druglike. (maximum is 4)
